# Elevated depressive symptoms in metabolic syndrome in a general population of Japanese men: a cross-sectional study

**DOI:** 10.1186/1471-2458-13-862

**Published:** 2013-09-18

**Authors:** Atsuko Sekita, Hisatomi Arima, Toshiharu Ninomiya, Tomoyuki Ohara, Yasufumi Doi, Yoichiro Hirakawa, Masayo Fukuhara, Jun Hata, Koji Yonemoto, Yukiko Ga, Takanari Kitazono, Shigenobu Kanba, Yutaka Kiyohara

**Affiliations:** 1 Department of Environmental Medicine, Graduate School of Medical Sciences, Kyushu University, 3-1-1 Maidashi, Higashi-ku, Fukuoka 812-8582, Japan; 2 Department of Neuropsychiatry, Graduate School of Medical Sciences, Kyushu University, 3-1-1 Maidashi, Higashi-ku, Fukuoka 812-8582, Japan; 3 Department of Medicine and Clinical Science, Graduate School of Medical Sciences, Kyushu University, 3-1-1 Maidashi, Higashi-ku, Fukuoka 812-8582, Japan; 4 Biostatistics Center, Kurume University, 67 Asahimachi, Kurume 830-0011, Japan

**Keywords:** Depressive symptoms, Metabolic syndrome, Population-based, Japanese

## Abstract

**Background:**

Uncertainty still surrounds the association between metabolic syndrome (MetS) and depression. We aimed to evaluate the association between MetS and elevated depressive symptoms in a general Japanese population.

**Methods:**

This is a cross-sectional survey of 3,113 community-dwelling individuals aged 40 years or over. MetS was defined according to the joint interim statement. MetS was diagnosed when a subject had three or more of the following components: 1) central obesity (waist circumference ≥90 cm for men, ≥80 cm in for women); 2) elevated blood pressure (≥130/85 mmHg or current use of antihypertensive medication); 3) hypertriglyceridemia (≥1.7 mmol/L); 4) low HDL cholesterol (< 1.0 mmol/L for men, < 1.3 mmol/L for women); and 5) elevated fasting plasma glucose (≥5.55 mmol/L or current use of antidiabetic medication). Depressive symptoms were assessed using the Center for Epidemiologic Studies Depression Scale (CES-D). The age- and multivariable-adjusted odds ratio (OR) and 95% confidence interval (CI) were estimated using a logistic regression model.

**Results:**

Elevated depressive symptoms were observed in 4.3% of male and 6.3% of female participants. In men, the age-adjusted prevalence of elevated depressive symptoms was significantly higher in subjects with MetS than in those without (7.1% versus 3.6%, p = 0.04). The prevalence of elevated depressive symptoms rose progressively as the number of MetS components increased (3.5%, 3.6%, 5.8%, and 9.2% in male subjects with 0–1, 2, 3, and ≥4 components, respectively; p = 0.02 for trend). This association remained significant even after adjustment for age, marital status, history of cardiovascular disease, smoking habit, alcohol intake, and regular exercise. In women, on the other hand, there was no clear association between MetS and depressive symptoms.

**Conclusions:**

MetS was associated with elevated depressive symptoms in a general population of Japanese men.

## Background

Depression is an important cause of long-term disability and dependency and is responsible for 11.8% of years-lived-with-disability [1,2]. Effective prevention of the burdens associated with depression will require a strategy based on better knowledge of its risk factors. Recently, a systematic review of observational studies demonstrated a link between metabolic syndrome (MetS) and depressive symptoms [3]. However, current knowledge of the association between MetS and depressive symptoms was derived mainly from studies conducted in Western populations; so it is unclear to what extent these findings apply to Asian populations. The present cross-sectional study evaluates the association of MetS with depressive symptoms in a general population of Japanese.

## Methods

### Study population and design

The Hisayama Study is a prospective cohort study of cerebro-cardiovascular diseases in a suburban community, the town of Hisayama, adjacent to of the city of Fukuoka, Japan [4-6]. Based on data from the national census, the age and occupational distributions in Hisayama have been almost identical to those in Japan as a whole since the 1960s [4]. As a part of this study, all 4330 residents of the town of Hisayama aged 40 years or older were invited to participate in a cross-sectional examination in 2007 and 2008. Among them, 3,376 residents consented to participate (participation rate 78.0%). After the exclusion of 263 subjects with missing data on depression or MetS, a total of 3,113 subjects were included in the present analysis.

### Metabolic syndrome

Information on current use of antihypertensive and antidiabetic medications was collected using a self-administered questionnaire and confirmed using the consumer drug information by trained staff. Blood pressure was measured three times after the subject had rested for at least 5 minutes prior to each measurement using a semiautomatic device (BP-203 RVIIIB; Omron Healthcare) based on the cuff-oscillometric principle with the subject in the sitting position. The mean of the three measurements was used for the analysis. Waist circumference was measured at the umbilical level in a standing position by a trained staff member. Blood samples were collected from an antecubital vein after an overnight fast for the determination of serum lipids and plasma glucose levels. Serum total cholesterol, triglyceride, and high-density lipoprotein (HDL) cholesterol concentrations were determined enzymatically. Fasting blood glucose levels were measured by the glucose oxidase method. MetS was defined based on the joint interim statement of the International Diabetes Federation Task Force on Epidemiology and Prevention; National Heart, Lung, and Blood Institute; American Heart Association; World Heart Federation; International Atherosclerosis Society; and International Association for the Study of Obesity [7]. MetS was diagnosed when a subject had three or more of the following components: 1) central obesity (waist circumference ≥90 cm for men, ≥80 cm for women); 2) elevated blood pressure (≥130/85 mmHg or current use of antihypertensive medication); 3) hypertriglyceridemia (≥1.7 mmol/L); 4) low HDL cholesterol (< 1.0 mmol/L for men, < 1.3 mmol/L for women); and 5) elevated fasting plasma glucose (≥5.55 mmol/L or current use of antidiabetic medication).

### Other covariates

Each participant completed a self-administered questionnaire covering marital status, medical history, smoking habit, alcohol intake, and exercise. The questionnaire was checked by trained interviewers. Marital status was classified as either having a spouse on a family register or not (e.g. single, divorced). A history of cardiovascular disease was defined as prior stroke (ICD10 codes I60, I61, I63 and I64) or coronary heart disease (I20 – I25) with or without coronary revascularization (coronary intervention or bypass surgery). A smoking habit was defined as current habitual smoking of 1 or more cigarettes per day. Alcohol intake was defined as current habitual drinking of at least once per month. Subjects who engaged in sports or other forms of exercise ≥3 times a week during their leisure time made up a regular exercise group.

### Elevated depressive symptoms

Depressive symptoms were assessed using the Japanese, 20-item version of the Center for Epidemiologic Studies Depression Scale (CES-D) [8,9], the reliability of which has been validated [10]. Elevated depressive symptoms were defined as a CES-D score of ≥16 or current use of antidepressant medication defined based on the self-administered questionnaire and the consumer drug information.

### Statistical analyses

In the present analysis, the key risk factor was MetS and its components, and the outcome was elevated depressive symptoms. All analyses were conducted separately for men and women. The prevalence of elevated depressive symptoms in each subgroup defined by MetS or its components was standardized for age distribution of the total study subjects by the direct method using 10-year age groupings. The association between components of Mets and elevated depressive symptoms was evaluated using an age-adjusted logistic regression model. The association of Mets and the number of its components with elevated depressive symptoms was evaluated using age- and multivariable-adjusted logistic regression models. The multivariable-adjusted models included age, marital status, history of cardiovascular disease, smoking habit, alcohol intake, and regular exercise as covariates. The differences in the association of MetS and the number of its components with elevated depressive symptoms between men and women were evaluated by adding interaction term(s) to the logistic regression models. P < 0.05 was considered statistically significant in all analyses. The SAS software package, version 9.2 (SAS Institute) was used for statistical analysis.

### Ethical considerations

The study protocol was approved by Kyushu University Institutional Review Board for Clinical Research, and the procedures followed were in accordance with national guidelines. All participants provided written informed consent.

## Results

The baseline characteristics of subjects with and without MetS are shown by sex in Table 1. Subjects with MetS had higher values for waist circumference, systolic and diastolic blood pressures, serum total cholesterol, triglycerides and fasting plasma glucose; they also had lower levels of HDL cholesterol in both men and women. The frequencies of antihypertensive and antidiabetic medications were also higher in MetS subjects of both sexes. Women with MetS were older and less likely to be alcohol drinkers.

**Table 1:** Characteristics of men and women with and without metabolic syndrome

	**Men**			**Women**		
	**MetS (−)**	**MetS (+)**	**P value**	**MetS (−)**	**MetS (+)**	**P value**
	**(n = 965)**	**(n = 388)**		**(n = 1261)**	**(n = 499)**	
Age (years)	63 ± 12	62 ± 10	0.14	62 ± 13	67 ± 11	< 0.0001
Single or divorced (%)	11.7	11.6	0.95	27.5	31.9	0.07
History of cardiovascular disease (%)	8.4	10.6	0.21	2.6	4.8	0.02
Antihypertensive medication (%)	28.2	44.6	< 0.0001	23.2	50.1	< 0.0001
Antidiabetic medication (%)	7.2	17.0	< 0.0001	1.8	17.6	< 0.0001
Waist circumference (cm)	83.5 ± 7.2	92.6 ± 6.8	< 0.0001	81.3 ± 9.3	91.4 ± 9.3	< 0.0001
Systolic blood pressure (mmHg)	129 ± 17	144 ± 15	< 0.0001	125 ± 18	144 ± 16	< 0.0001
Diastolic blood pressure (mmHg)	79 ± 9	88 ± 9	< 0.0001	75 ± 10	84 ± 10	< 0.0001
Total cholesterol (mmol/L)	5.10 ± 0.87	5.34 ± 0.93	0.0001	5.57 ± 0.89	5.73 ± 0.93	0.0008
Triglycerides (mmol/L)	1.10(0.82–1.47)	2.02(1.47–2.84)	< 0.0001	0.93(0.69–1.23)	1.57(1.09–2.04)	< 0.0001
HDL cholesterol (mmol/L)	1.65 ± 0.42	1.39 ± 0.37	< 0.0001	1.95 ± 0.43	1.61 ± 0.42	< 0.0001
Fasting plasma glucose (mmol/L)	5.8 ± 1.2	6.7 ± 1.6	< 0.0001	5.3 ± 0.7	6.4 ± 1.5	< 0.0001
Smoking habit (%)	33.6	38.0	0.12	7.6	7.3	0.82
Alcohol intake (%)	68.0	71.4	0.22	33.7	26.7	0.004
Regular exercise (%)	13.7	12.9	0.70	10.1	12.6	0.12

*MetS* metabolic syndrome; *HDL* high-density lipoprotein.

Values are mean ± SD, median (interquartile range) or frequency.

The differences between subjects with and without MetS were tested by Wilcoxon tests for continuous variables and chi-square tests for categorical variables.

Elevated depressive symptoms were observed in 58 (4.3%) men and 111 (6.3%) women. Table 2 shows the age-adjusted prevalence and odds ratio [OR] for elevated depressive symptoms according to the presence of MetS components in men and women. The age-adjusted prevalence of elevated depressive symptoms was higher in men with low HDL cholesterol (age-adjusted OR 2.55 [95% CI 1.11-5.86]) and men with elevated fasting plasma glucose (1.90 [95% CI 1.06-3.42]). These associations remained significant even after controlling for age, marital status, history of cardiovascular disease, smoking habit, alcohol intake, and regular exercise (low HDL cholesterol: multivariable-adjusted OR 2.44 [95% CI 1.04-5.69], elevated fasting plasma glucose: 1.93 [95% CI 1.07-3.49]). In women, on the other hand, there were no clear associations between MetS components and depressive symptoms (all p > 0.05).

**Table 2:** Components of metabolic syndrome and elevated depressive symptoms in men and women

	**N of cases/participants**	**Age-adjusted prevalence (%)**	**Age-adjusted OR (95% CI)**	**P value**
Men				
Central obesity				
No	34/912	3.6	Reference	
Yes	24/441	5.9	1.61 (0.94–2.77)	0.08
Elevated BP				
No	21/582	3.9	Reference	
Yes	37/771	4.9	1.26 (0.73–2.18)	0.41
Hypertriglyceridemia				
No	40/939	4.1	Reference	
Yes	18/414	4.8	1.17 (0.66–2.09)	0.60
Low HDL cholesterol				
No	51/1276	4.0	Reference	
Yes	7/77	8.3	2.55 (1.11–5.86)	0.03
Elevated FPG				
No	16/570	2.9	Reference	
Yes	42/783	5.5	1.90 (1.06–3.42)	0.03
Women				
Central obesity				
No	43/593	7.4	Reference	
Yes	68/1167	5.9	0.78 (0.53–1.16)	0.22
Elevated BP				
No	58/899	6.8	Reference	
Yes	53/861	6.2	0.91 (0.61–1.36)	0.65
Hypertriglyceridemia				
No	100/1471	6.8	Reference	
Yes	11/289	3.6	0.54 (0.28–1.01)	0.05
Low HDL cholesterol				
No	102/1607	6.3	Reference	
Yes	9/153	6.4	0.92 (0.45–1.85)	0.81
Elevated FPG				
No	71/1103	6.5	Reference	
Yes	40/657	6.3	0.93 (0.62–1.39)	0.71

*OR* odds ratio, 95% *CI* 95% confidence interval, *BP* blood pressure, *HDL* high-density lipoprotein, *FPG* fasting plasma glucose.

OR and P values were estimated using logistic regression models.

Table 3 shows the association of MetS with depressive symptoms in men and women. The age-adjusted prevalence of elevated depressive symptoms was significantly higher in men with MetS than in those without it (7.1% versus 3.6%; age-adjusted OR 1.78 [95% CI 1.03-3.08]). This association remained significant even after controlling for age, marital status, history of cardiovascular disease, smoking habit, alcohol intake, and regular exercise (multivariable-adjusted OR 1.82 [95% CI 1.05-3.15]). In women, on the other hand, there were no significant associations between MetS and depressive symptoms (p = 0.10; p = 0.006 for homogeneity between men and women).

**Table 3:** Metabolic syndrome and elevated depressive symptoms in men and women

	**N of cases/participants**	**Age-adjusted**			Multivariable-adjusted^*^	
		**Prevalence (%)**	**OR (95% CI)**	**P value**	**OR (95% CI)**	**P value**
Men						
MetS (−)	35/965	3.6	Reference		Reference	
MetS (+)	23/388	7.1	1.78 (1.03–3.08)	0.04	1.82 (1.05–3.15)	0.03
Women						
MetS (−)	87/1261	7.0	Reference		Reference	
MetS (+)	24/499	5.2	0.66 (0.41–1.06)	0.08	0.67 (0.42–1.08)	0.10

*MetS* metabolic syndrome, *OR* odds ratio, *95%CI* 95% confidence interval.

OR and P values were estimated using logistic regression models.

^*^Adjusted for age, marital status, history of cardiovascular disease, smoking habit, alcohol intake and regular exercise.

The age-adjusted prevalence and adjusted OR for elevated depressive symptoms according to the number of MetS components are shown by sex in Table 4. In men, the age-adjusted prevalence rose with the number of MetS components: it was 3.5%, 3.6%, 5.8%, and 9.2% for subjects with 0–1, 2, 3, and ≥4 components, respectively (p = 0.02 for trend). A significant association was observed even after controlling for the aforementioned confounding factors (p = 0.01 for trend). When the number of MetS was used as a continuous variable, an increase in 1 component was associated with a 35% (95% CI 8-67%) increase in elevated depressive symptoms. In contrast to men, women showed no clear associations between the number of MetS components and depressive symptoms (p = 0.17 for trend; OR 0.9 (95% CI 0.76-1.06) per 1 component increase; p = 0.04 for homogeneity between men and women).

**Table 4:** The number of metabolic syndrome components and elevated depressive symptoms in men and women

	**N of cases/participants**	**Age-adjusted**			Multivariable-adjusted*	
**Number of MetS components**		**Prevalence (%)**	**OR (95% CI)**	**P trend**	**OR (95% CI)**	**P trend**
Men						
0-1	20/567	3.5	Reference		Reference	
2	15/398	3.6	0.80 (0.44–1.45)		0.84 (0.46–1.55)	
3	12/250	5.8	1.17 (0.61–2.25)		1.20 (0.62–2.31)	
≥4	11/138	9.2	2.43 (1.22–4.86)	0.02	2.47 (1.22–4.96)	0.01
Women						
0-1	56/782	7.4	Reference		Reference	
2	31/479	6.3	1.03 (0.67–1.58)		1.05 (0.68–1.62)	
3	15/360	4.4	0.58 (0.33–1.01)		0.56 (0.32–0.99)	
≥4	9/139	7.5	1.01 (0.50–2.06)	0.12	1.10 (0.54–2.25)	0.17

*MetS* metabolic syndrome, *OR* odds ratio, *95%CI* 95% confidence interval.

OR and P values were estimated using logistic regression models.

*Adjusted for age, marital status, history of cardiovascular disease, smoking habit, alcohol intake and regular exercise.

## Discussion

The present cross-sectional examination of a general population of Japanese demonstrated a higher prevalence of elevated depressive symptoms in male subjects with MetS compared to those without it. This association remained significant even after controlling for the effects of age, marital status, history of cardiovascular disease, smoking habit, alcohol intake, and regular exercise. Furthermore, the prevalence of elevated depressive symptoms rose with the number of MetS components. In female subjects, on the other hand, there was no clear association between MetS and depressive symptoms.

Although a number of observational studies have investigated the association between MetS and depressive symptoms, their conclusions have been inconsistent [11-29]. With regard to cross-sectional studies, a French study demonstrated elevated depressive symptoms in men and women with MetS [14]. The PPP-Bonita Study also showed close associations of MetS and its components with depressive symptoms in Finnish men and women [29]. With respect to longitudinal design research, the Whitehall II study demonstrated that the presence of MetS was associated with 38% increased risks of future depressive symptoms in men and women in London [16]. The Health in Men Study showed that MetS was a strong predictor of the future development of depression in elderly Australian men [17]. A positive association between MetS and the incidence of depression was also reported in an office-based study of 956 Japanese men [18]. On the other hand, a cohort study from France demonstrated no significant association between MetS and depressive symptoms in elderly subjects aged about 70–90 years old [24]. A recent systematic review and a meta analysis including all these studies, however, demonstrated a clear relationship between MetS and depression. Our findings from the Hisayama Study suggest that the concept of a link between MetS and depression is likely to be applicable to Japanese men.

Several population-based observational studies have reported the association between MetS and depression separately for men and women. A cross-sectional study in France demonstrated that depression and depressive symptoms were associated with MetS, irrespective of gender [14]. A cross-sectional study in Poland showed that MetS was observed more frequently among male subjects with depressive symptoms than those without, while there were no associations among women [12]. A cross-sectional study in the United States reported that women with a history of major depressive episode were twice as likely to have metabolic syndrome compared with those without such a history, but men with history of depression were not significantly more likely to have MetS [11]. A cohort study in Finland found that MetS was not associated with depression or anxiety in either men or women [13]. Therefore, there has been significant inconsistency in gender differences in the link between MetS and depression in Western populations. With regard to Asian populations, on the other hand, a cohort study of Japanese male employees showed a positive relationship between MetS and depression [18]. A cross-sectional study of Japanese subjects in Takarazuka City demonstrated that the mean depression score was higher for men with MetS than those without it, while depression was not associated with MetS in women [25]. In the present study of Japanese subjects, MetS was associated with elevated depressive symptoms in men but not in women. In Asian populations, MetS may be associated with depressive symptoms only in men.

One of the mechanisms underlying the association between MetS and depressive symptoms is thought to be the stress-induced hyperactivity of the hypothalamic-pituitary-adrenal (HPA) axis, which is common in depression [30-32] and could lead to metabolic alterations [33-35]. Chronic stress has also been shown to increase the risk of metabolic disorders through elevated sympathetic activity [36]. Another potential mechanism is that cerebral small vessel disease associated with MetS [37] can increase the risk of late-life depression [38,39]. It is also possible that behavioral factors associated with depressive symptoms, such as physical inactivity and poor diet, contribute to central adiposity and metabolic disorders.

Another important finding from the present analysis is the lack of associations between MetS and depressive symptoms in women. This finding is consistent with a previous cross-sectional study. The SOPKARD project demonstrated clear associations between MetS and depressive symptoms in Polish men but not in Polish women [12]. The reason for this discrepancy has not been clearly resolved, but it may be attributable to heterogeneity in genetic factors, hormonal factors, socio-economic factors and social roles between men and women.

There were several limitations to this study. Because of the cross-sectional nature of the study, we were unable to determine whether or not there was a causal association between MetS and the development of depressive symptoms. In addition, we were unable to address the potential mechanisms underlying the reported associations, the self-reported covariates had somewhat limited accuracy, and the study lacked definite diagnosis of depression based on structured interviews with psychiatrists using standard criteria.

## Conclusions

In conclusion, MetS was associated with elevated depressive symptoms in a general population of Japanese men. Screening of depressive symptoms in men with MetS is likely to aid in the early detection and treatment of depression, and might be able to provide additional protection against the enormous burden of mental disorders in Japan.

## Abbreviations

MetS: Metabolic syndrome; CES-D: Center for epidemiologic studies depression scale; HDL: High-density lipoprotein; OR: Odds ratio; CI: Confidence interval; HPA: Hypothalamic-pituitary-adrenal.

## Competing interests

The authors declare that they have no competing interests.

## Authors’ contributions

AS conducted the statistical analyses, wrote the manuscript, and reviewed and edited the manuscript. AS is the guarantor of this work, had full access to all data in the study and takes responsibility for the integrity of the data and the accuracy of the data analysis. HA contributed to discussion and reviewed and edited the manuscript. TN, TO and YD contributed to discussion. YH brushed up the accuracy of data. MF, JH, KY, YG, TK and SK contributed to discussion. YK is responsible for overall management of the Hisayama Study, and reviewed and edited the manuscript. All authors read and approved the final manuscript.

## Pre-publication history

The pre-publication history for this paper can be accessed here:

http://www.biomedcentral.com/1471-2458/13/862/prepub

## References

[B1] Murray CJL Lopez AP Global burden of disease: a comprehensive assessment of mortality and disability from diseases, injuries, and risk factors in 1990 and projected to 2020 1996 Cambridge: Harvard University Press

[B2] Prince M Patel V Saxena S Maj M Maselko J Phillips MR Rahman A No health without mental health Lancet 2007 370 859 877 10.1016/S0140-6736(07)61238-0 17804063

[B3] Pan A Keum N Okereke OI Sun Q Kivimaki M Rubin RR Hu FB Bidirectional association between depression and metabolic syndrome: a systematic review and meta-analysis of epidemiological studies Diabetes Care 2012 35 1171 1180 10.2337/dc11-2055 22517938 PMC3329841

[B4] Ohmura T Ueda K Kiyohara Y Kato I Iwamoto H Nakayama K Nomiyama K Ohmori S Yoshitake T Shinkawu A Prevalence of type 2 (non-insulin-dependent) diabetes mellitus and impaired glucose tolerance in the Japanese general population: the Hisayama study Diabetologia 1993 36 1198 1203 10.1007/BF00401066 8270136

[B5] Arima H Kubo M Yonemoto K Doi Y Ninomiya T Tanizaki Y Hata J Matsumura K Iida M Kiyohara Y High-sensitivity C-reactive protein and coronary heart disease in a general population of Japanese: the Hisayama study Arterioscler Thromb Vasc Biol 2008 28 1385 1391 10.1161/ATVBAHA.107.157164 18403728

[B6] Sekita A Ninomiya T Tanizaki Y Doi Y Hata J Yonemoto K Arima H Sasaki K Iida M Iwaki T Trends in prevalence of Alzheimer’s disease and vascular dementia in a Japanese community: the Hisayama study Acta Psychiatr Scand 2010 122 319 325 10.1111/j.1600-0447.2010.01587.x 20626720

[B7] Alberti KG Eckel RH Grundy SM Zimmet PZ Cleeman JI Donato KA Fruchart JC James WP Loria CM Smith SC Jr Harmonizing the metabolic syndrome: a joint interim statement of the international diabetes federation task force on epidemiology and prevention; national heart, lung, and blood institute; american heart association; world heart federation; international atherosclerosis society; and international association for the study of obesity Circulation 2009 120 1640 1645 10.1161/CIRCULATIONAHA.109.192644 19805654

[B8] Radloff LS The CES-D scale: a self-report depression scale for research in the general population Applied Psychol Measure 1977 1 385 401 10.1177/014662167700100306

[B9] Roberts RE Vernon SW The center for epidemiologic studies depression scale: its use in a community sample Am J Psychiatry 1983 140 41 46 6847983 10.1176/ajp.140.1.41

[B10] Sima S Kano T Kitamura T Asai M A new scale of depressive symptoms (in Japanese) Clinical Psychiatry 1985 27 717 723

[B11] Kinder LS Carnethon MR Palaniappan LP King AC Fortmann SP Depression and the metabolic syndrome in young adults: findings from the third national health and nutrition examination survey Psychosom Med 2004 66 316 322 10.1097/01.psy.0000124755.91880.f4 15184689

[B12] Gil K Radzillowicz P Zdrojewski T Pakalska-Korcala A Chwojnicki K Piwonski J Ignaszewska-Wyrzykowska A Zaluga L Mielczarek M Landowski J Relationship between the prevalence of depressive symptoms and metabolic syndrome. Results of the SOPKARD project Kardiol Pol 2006 64 464 469 16752328

[B13] Herva A Rasanen P Miettunen J Timonen M Laksy K Veijola J Laitinen J Ruokonen A Joukamaa M Co-occurrence of metabolic syndrome with depression and anxiety in young adults: the Northern Finland 1966 birth cohort study Psychosom Med 2006 68 213 216 10.1097/01.psy.0000203172.02305.ea 16554385

[B14] Skilton MR Moulin P Terra JL Bonnet F Associations between anxiety, depression, and the metabolic syndrome Biol Psychiatry 2007 62 1251 1257 10.1016/j.biopsych.2007.01.012 17553465

[B15] Vogelzangs N Suthers K Ferrucci L Simonsick EM Ble A Schrager M Bandinelli S Lauretani F Giannelli SV Penninx BW Hypercortisolemic depression is associated with the metabolic syndrome in late-life Psychoneuroendocrinol 2007 32 151 159 10.1016/j.psyneuen.2006.11.009 PMC1868465 17224244

[B16] Akbaraly TN Kivimaki M Brunner EJ Chandola T Marmot MG Singh-Manoux A Ferrie JE Association between metabolic syndrome and depressive symptoms in middle-aged adults: results from the Whitehall II study Diabetes Care 2009 32 499 504 10.2337/dc08-1358 19106378 PMC2646036

[B17] Almeida OP Calver J Jamrozik K Hankey GJ Flicker L Obesity and metabolic syndrome increase the risk of incident depression in older men: the health in men study Am J Geriatr Psychiatry 2009 17 889 898 10.1097/JGP.0b013e3181b047e3 19910877

[B18] Takeuchi T Nakao M Nomura K Inoue M Tsurugano S Shinozaki Y Yano E Association of the metabolic syndrome with depression and anxiety in Japanese men: a 1-year cohort study Diabetes Metab Res Rev 2009 25 762 767 10.1002/dmrr.1041 19839027

[B19] Vanhala M Jokelainen J Keinanen-Kiukaanniemi S Kumpusalo E Koponen H Depressive symptoms predispose females to metabolic syndrome: a 7-year follow-up study Acta psychiatr Scand 2009 119 137 142 10.1111/j.1600-0447.2008.01283.x 19016666

[B20] Vogelzangs N Beekman AT Dik MG Bremmer MA Comijs HC Hoogendijk WJ Deeg DJ Penninx BW Late-life depression, cortisol, and the metabolic syndrome Am J Geriatr Psychiatry 2009 17 716 721 10.1097/JGP.0b013e3181aad5d7 19625789

[B21] East C Willis BL Barlow CE Grannemann BD FitzGerald SJ DeFina LF Trivedi MH Depressive symptoms and metabolic syndrome in preventive healthcare: the cooper center longitudinal study Metab Syndr Relat Disord 2010 8 451 457 10.1089/met.2010.0017 20854094

[B22] Kobrosly RW van Wijngaarden E Revisiting the association between metabolic syndrome and depressive symptoms Ann Epidemiol 2010 20 852 855 10.1016/j.annepidem.2010.08.002 20933192

[B23] Lamers F de Jonge P Nolen WA Smit JH Zitman FG Beekman AT Penninx BW Identifying depressive subtypes in a large cohort study: results from the Netherlands study of depression and anxiety (NESDA) J Clin Psychiatry 2010 71 1582 1589 10.4088/JCP.09m05398blu 20673552

[B24] Akbaraly TN Ancelin ML Jaussent I Ritchie C Barberger-Gateau P Dufouil C Kivimaki M Berr C Ritchie K Metabolic syndrome and onset of depressive symptoms in the elderly: findings from the three-city study Diabetes Care 2011 34 904 909 10.2337/dc10-1644 21346185 PMC3064049

[B25] Nishina M Nishina K Ohira T Makino K Iso H Associations of psychological distress with metabolic syndrome among Japanese urban residents J Atheroscler Thromb 2011 18 396 402 10.5551/jat.6692 21325777

[B26] Vogelzangs N Beekman AT Boelhouwer IG Bandinelli S Milaneschi Y Ferrucci L Penninx BW Metabolic depression: a chronic depressive subtype? Findings from the InCHIANTI study of older persons J Clin Psychiatry 2011 72 598 604 10.4088/JCP.10m06559 21535996 PMC6232848

[B27] Kahl KG Greggersen W Schweiger U Cordes J Balijepalli C Losch C Moebus S Prevalence of the metabolic syndrome in unipolar major depression Eur Arch Psychiatry Clin Neurosci 2012 262 313 320 10.1007/s00406-011-0277-4 22183567

[B28] Seppala J Vanhala M Kautiainen H Eriksson J Kampman O Mantyselka P Oksa H Ovaskainen Y Viikki M Koponen H Prevalence of metabolic syndrome in subjects with melancholic and non-melancholic depressive symptoms. A Finnish population-based study J Affect Disord 2012 136 543 549 10.1016/j.jad.2011.10.032 22119085

[B29] Pyykkonen AJ Raikkonen K Tuomi T Eriksson JG Groop L Isomaa B Association between depressive symptoms and metabolic syndrome is not explained by antidepressant medication: results from the PPP-Botnia Study Ann Med 2012 44 279 288 10.3109/07853890.2010.543921 21254903

[B30] Nemeroff CB Widerlov E Bissette G Walleus H Karlsson I Eklund K Kilts CD Loosen PT Vale W Elevated concentrations of CSF corticotropin-releasing factor-like immunoreactivity in depressed patients Science 1984 226 1342 1344 10.1126/science.6334362 6334362

[B31] Wolkowitz OM Reus VI Manfredi F Ingbar J Brizendine L Weingartner H Ketoconazole administration in hypercortisolemic depression Am J Psychiatry 1993 150 810 812 8480829 10.1176/ajp.150.5.810

[B32] Jabben N Nolen WA Smit JH Vreeburg SA Beekman AT Penninx BW Co-occurring manic symptomatology influences HPA axis alterations in depression J Psychiatr Res 2011 45 1208 1213 21450306 10.1016/j.jpsychires.2011.03.010

[B33] Rosmond R Role of stress in the pathogenesis of the metabolic syndrome Psychoneuroendocrinol 2005 30 1 10 10.1016/j.psyneuen.2004.05.007 15358437

[B34] Anagnostis P Athyros VG Tziomalos K Karagiannis A Mikhailidis DP Clinical review: the pathogenetic role of cortisol in the metabolic syndrome: a hypothesis J Clin Endocrinol Metab 2009 94 2692 2701 10.1210/jc.2009-0370 19470627

[B35] Tentolouris N Argyrakopoulou G Katsilambros N Perturbed autonomic nervous system function in metabolic syndrome Neuromolecular Med 2008 10 169 178 10.1007/s12017-008-8022-5 18224460

[B36] Licht CM Vreeburg SA van Reedt Dortland AK Giltay EJ Hoogendijk WJ DeRijk RH Vogelzangs N Zitman FG de Geus EJ Penninx BW Increased sympathetic and decreased parasympathetic activity rather than changes in hypothalamic-pituitary-adrenal axis activity is associated with metabolic abnormalities J Clin Endocrinol Metab 2010 95 2458 2466 10.1210/jc.2009-2801 20237163

[B37] Bokura H Yamaguchi S Iijima K Nagai A Oguro H Metabolic syndrome is associated with silent ischemic brain lesions Stroke 2008 39 1607 1609 10.1161/STROKEAHA.107.508630 18323475

[B38] Herrmann LL Le Masurier M Ebmeier KP White matter hyperintensities in late life depression: a systematic review J Neurol Neurosurg Psychiatry 2008 79 619 624 17717021 10.1136/jnnp.2007.124651

[B39] Santos M Gold G Kovari E Herrmann FR Bozikas VP Bouras C Giannakopoulos P Differential impact of lacunes and microvascular lesions on poststroke depression Stroke 2009 40 3557 3562 10.1161/STROKEAHA.109.548545 19696424

